# In Silico Deciphering of the Potential Impact of Variants of Uncertain Significance in Hereditary Colorectal Cancer Syndromes

**DOI:** 10.3390/cells13161314

**Published:** 2024-08-06

**Authors:** Candida Fasano, Martina Lepore Signorile, Katia De Marco, Giovanna Forte, Vittoria Disciglio, Paola Sanese, Valentina Grossi, Cristiano Simone

**Affiliations:** 1 Medical Genetics, National Institute of Gastroenterology, IRCCS “Saverio de Bellis” Research Hospital, 70013 Castellana Grotte, Italy; martina.lepore@irccsdebellis.it (M.L.S.); katia.demarco@irccsdebellis.it (K.D.M.); giovanna.forte@irccsdebellis.it (G.F.); vittoria.disciglio@irccsdebellis.it (V.D.); paola.sanese@irccsdebellis.it (P.S.); valentina.grossi@irccsdebellis.it (V.G.); 2 Medical Genetics, Department of Precision and Regenerative Medicine and Jonic Area (DiMePRe-J), University of Bari Aldo Moro, 70124 Bari, Italy

**Keywords:** hereditary colorectal polyposis syndromes, hereditary nonpolyposis colorectal cancer, variants of uncertain significance, in silico prediction tools, protein stability, protein functions

## Abstract

Colorectal cancer (CRC) ranks third in terms of cancer incidence worldwide and is responsible for 8% of all deaths globally. Approximately 10% of CRC cases are caused by inherited pathogenic mutations in driver genes involved in pathways that are crucial for CRC tumorigenesis and progression. These hereditary mutations significantly increase the risk of initial benign polyps or adenomas developing into cancer. In recent years, the rapid and accurate sequencing of CRC-specific multigene panels by next-generation sequencing (NGS) technologies has enabled the identification of several recurrent pathogenic variants with established functional consequences. In parallel, rare genetic variants that are not characterized and are, therefore, called variants of uncertain significance (VUSs) have also been detected. The classification of VUSs is a challenging task because each amino acid has specific biochemical properties and uniquely contributes to the structural stability and functional activity of proteins. In this scenario, the ability to computationally predict the effect of a VUS is crucial. In particular, in silico prediction methods can provide useful insights to assess the potential impact of a VUS and support additional clinical evaluation. This approach can further benefit from recent advances in artificial intelligence-based technologies. In this review, we describe the main in silico prediction tools that can be used to evaluate the structural and functional impact of VUSs and provide examples of their application in the analysis of gene variants involved in hereditary CRC syndromes.

## 1. Introduction

Colorectal cancer (CRC) is the third most common cancer in the world and accounts for more than 8% of deaths from all causes annually [1]. Between 6 and 10% of all CRC cases and around 20% of those detected before the age of 50 have identifiable hereditary pathogenic mutations in genes that significantly increase CRC susceptibility [2,3]. In most hereditary CRC syndromes, cancer arises from primary lesions such as polyps and adenomas, but the pathways leading to carcinoma development vary in the different disorders. The identification of the main hereditary mutations involved in these disorders has been crucial to improving the comprehension of the basic molecular processes responsible for CRC tumorigenesis [4]. Genetic susceptibility to CRC seems more widespread than previously expected. Recent reports uncovered disease-causing genetic variants in a wide variety of cancer susceptibility genes with high and moderate penetrance [5]. These pathogenic variants have been described in over 10% of patients diagnosed with advanced cancer, including CRC [6]. 

The advent of next-generation sequencing (NGS) technologies has significantly enhanced our ability to identify genetic variants. In addition to expediting the identification of recurrent pathogenic variants with established functional consequences, NGS has also revealed several rare uncharacterized genetic variants, which are, therefore, called variants of uncertain significance (VUSs) [7]. The majority of VUSs can be grouped into three main categories based on the type of genetic alteration, i.e., missense substitutions (most frequent), splice junction variants, and in-frame insertion or deletion variants (in-frame indels), but their functional classification has proven challenging when using multigene panels in genetic testing [4,8]. The assessment of the functional impact of a missense VUS is complex due to the specific biochemical properties of each amino acid, which modify the stability and function of the affected protein. Therefore, a missense substitution can have a variety of effects, ranging from no impact to completely abolishing protein function or even leading to the acquisition of new functions or increased stability. Splice junction variants can abrogate splicing or increase or decrease its efficiency. In particular, they can affect precursor mRNA-spliceosome interactions, leading to exon skipping, full intron inclusion, and alternative use of neighboring cryptic splice sites [9]. These events result in nucleotide insertions or deletions (in-frame indels) that impair protein structure and function due to extra or missing amino acids or even entire domains [10].

VUS assessment is particularly important when the variant occurs in a clinically significant gene, as the interpretation of its structural and functional implications can be very useful in clinical practice and for the surveillance of hereditary disorders [11]. Clinicians, therefore, need clear guidance regarding the significance of variants that may have practical consequences. Following genetic testing to detect mutations in germline CRC susceptibility genes, three possible outcomes can occur: (i) no variant is found; (ii) the identified variant is known as pathogenic or benign; or (iii) the identified variant is a VUS. If a pathogenic variant is detected, the patient should receive genetic counseling and be treated according to gene-specific guidelines and their personal and family history of cancer. Moreover, “cascade testing” should be performed on relatives at risk to ascertain whether they also carry the variant, and appropriate screening programs should be recommended, including earlier and more frequent colonoscopies [12,13].

According to the National Comprehensive Cancer Network (NCCN, https://www.nccn.org, accessed on 5 July 2024) guidelines, clinical surveillance for patients with a VUS in an oncogene associated with hereditary CRC syndromes should be the same as that indicated for the general population [14]. Still, in these cases, the clinical geneticist is responsible for evaluating the patient’s clinical phenotype and family history to decide whether a segregation analysis of the VUS in the family is warranted. Although VUSs are not used as markers to increase clinical surveillance, it should be noted that many of the variants originally classified as VUSs have been subsequently characterized as pathogenic, thus initially escaping NCCN-recommended clinical surveillance programs, with serious clinical implications for the affected patients. Therefore, the discovery of a VUS poses a problem because it is unclear if the mutation is benign or pathogenic, and family members cannot be stratified according to their risk of developing CRC. This makes clinical management more challenging. Clinicians can only evaluate the putative functional implications of a VUS based on information gathered from specific databases, which unfortunately are not updated on a regular basis, and current literature [15]. These limitations may be overcome, at least in part, by the use of in silico tools. This approach can provide valuable insights by predicting the potential impact of the identified variants on protein structure and function. As such, in silico tools are crucial resources for prioritizing specific VUSs for further investigation and guiding clinical decisions [11]. 

Ideally, the management of genetic disorders associated with CRC would require collaborative efforts from multidisciplinary teams to integrate computational predictions with experimental validations and genetic counseling. These three key aspects are essential for enhancing the accuracy of VUS interpretation and promoting more efficient clinical surveillance, with the ultimate goal of advancing personalized medicine. 

In this review, we summarize current in silico methodologies available to assess the structural and functional implications of VUSs in key genes playing a role in hereditary CRC syndromes. 

## 2. Pathology of Hereditary CRC Syndromes

CRC is an epithelial-originated cancer that typically begins as an adenoma. While the majority of CRCs occur in individuals with no family history of the disease or other risk conditions, approximately 30% of CRC patients have family members affected by the same cancer [16]. Current epidemiological evidence shows that people are more likely to develop CRC or adenomatous polyps if they have one or more first-degree relatives affected by these conditions. Although not fully clear yet, this may be due to a mix of shared environmental variables and genetic factors [17]. 

CRC screening guidelines recommend that most average-risk patients start screening at 50 years of age [18]. The suggested screening age and frequency may vary based on the presence of polyps with specific histotypes or a family history of CRC. Patients who have a first-degree relative diagnosed with CRC and a family history of the disease should have a colonoscopy every 5 years beginning at 40 years of age or 10 years before their relative’s diagnosis age [18,19]. 

Hereditary CRC syndromes are associated with a significant increase in CRC risk and early onset of the disease. Based on the number and histotype of CRC lesions, they can be classified into two major phenotypic categories: polyposis and nonpolyposis syndromes [1]. 

### 2.1. Hereditary CRC Polyposis Syndromes

Hereditary CRC polyposis syndromes comprise familial adenomatous polyposis (FAP), attenuated familial adenomatous polyposis (AFAP), MUTYH-associated polyposis (MAP), polymerase proofreading-associated polyposis (PPAP), NTHL1 tumor syndrome, Peutz–Jeghers syndrome (PJS), juvenile polyposis syndrome (JPS), PTEN hamartoma tumor syndrome (PHTS), hereditary mixed polyposis syndrome (HMPS), serrated polyposis syndrome (SPS), and the recently characterized gastric polyposis and desmoid FAP (GD-FAP) [1,20].

FAP is an autosomal dominant hereditary cancer syndrome caused by germline heterozygous mutations in the adenomatous polyposis coli (*APC*) gene, which is located on chromosome 5q21 and is considered the ‘gatekeeper’ tumor suppressor gene for CRC [21,22]. This condition is the second most prevalent inherited CRC syndrome, representing about 1% of all CRC cases [1]. FAP is characterized by the early (late childhood) appearance of hundreds to thousands of adenomatous polyps [22]. In patients with FAP, the development of CRC depends on the co-occurrence of two molecular events triggering the disease, as postulated by Knudson’s two-hit hypothesis. The first is a germline *APC* mutation, and the second may be an additional somatic mutation in *APC* or its loss of heterozygosity (LOH) [23]. Although further mutations in the *KRAS*, *TP53*, and *SMAD4* genes may occur during FAP-related tumorigenesis, *APC* loss or germline mutations are crucial steps triggering CRC [24,25]. FAP genotypes are further complicated by the presence of several VUSs in the *APC* gene. 

AFAP is a subtype of FAP in which patients develop less severe symptoms. AFAP patients exhibit fewer than 100 polyps, delayed initiation of colorectal adenomas, and a likely lower lifetime risk of CRC. In these patients, adenomas are often flat and located in the proximal colonic region and upper gastrointestinal tract [26]. Approximately 10% of AFAP patients display mutations in exon 9 as well as in the 5′ and 3′ terminal regions of the *APC* gene. Additionally, 7% of these patients have a genetic alteration in the *MUTYH* gene [27]. Based on the annotations recorded in the ClinVar Miner database (https://clinvarminer.genetics.utah.edu/, accessed on 7 April 2024 [28]), out of 10,625 *APC* variants associated with FAP and AFAP syndromes, 6139 are VUSs (accessed on 7 April 2024) (Table 1).

GD-FAP was recently described as a novel FAP clinical variant characterized by widespread gastric polyposis and the presence of desmoid tumors as extracolonic lesions. Genetically, GD-FAP patients exhibit germline mutations in the extreme 3′ end of the *APC* gene [20].

MAP is an autosomal recessive syndrome caused by biallelic germline variants in the *MUTYH* gene, which encodes a central effector of the DNA base excision repair (BER) pathway involved in oxidative stress response [29]. Patients with MAP show a phenotype that mimics FAP and AFAP syndromes, ranging from one colorectal adenocarcinoma and a few polyps to serrated polyps [30]. Monoallelic *MUTYH* mutations have been linked to a higher risk of CRC, particularly in MAP patients with first-degree relatives who had the disease [31]. Based on current ClinVar Miner data, 14 out of 51 genetic variants identified so far in the *MUTYH* gene are classified as VUSs.

PPAP is an autosomal dominant polyposis syndrome characterized by germline heterozygous missense variants located in the exonuclease (proofreading) domains of the polymerase-coding genes *POLE* or *POLD* [32]. PPAP patients may exhibit FAP or AFAP phenotypes along with other tumors showing somatic hypermutation [33]. Based on the annotations reported in the ClinVar Miner database, 2378 VUSs have been identified in the *POLD* gene and 379 in the *POLE* gene (Table 1).

NTHL1 tumor syndrome, a recently identified rare autosomal recessive polyposis, is caused by biallelic variations in the *NTHL1* gene. NTHL1 is a DNA N-glycosylase that catalyzes the first step of the BER pathway [34,35]. Patients with NTHL1 tumor syndrome exhibit many tumors, all clinically associated with polyposis [35]. To date, 201 *NTHL1* germline variants have been associated with NTHL1 tumor syndrome, more than half of which (102) are VUSs (Table 1). 

Hamartomatous polyposis syndromes (HPS) are a subtype of CRC polyposis that exhibit autosomal dominant patterns of inheritance and include PJS, JPS, and PHTS [36]. 

PJS is caused by germline mutations in the tumor suppressor serine-threonine kinase STK11 gene (*STK11*) and is often associated with autosomal dominant mutations in the serine/threonine-protein kinase MTOR gene (*MTOR*) [37,38]. STK11 regulates cell proliferation, metabolism, and cell polarity [39,40]. Germline pathogenic *STK11* mutations are detected in 50–70% of PJS patients [4]. Clinically, the appearance of PJS polyps occurs early, at an average age of 12 years. PJS patients may develop a variable number of polyps located exclusively in the small intestine and often exhibit mucocutaneous pigmentations and a family history of PJS [36]. Of note, out of 1912 germline *STK11* mutations that have been associated with PJS, 837 are VUSs.

JPS is defined by the presence of several colonic and/or stomach hamartomas. Approximately 50–70% of JPS patients have been shown to harbor germline pathogenic mutations in the *BMPR1A* and *SMAD4* genes. JPS is linked to a high risk of gastric and colorectal malignancies. People with *SMAD4* mutations have an increased likelihood of developing hereditary hemorrhagic telangiectasia (HHT) [36]. Based on ClinVar Miner annotations, 1600 germline *BMPR1A* variants and 1348 germline *SMAD4* variants are associated with JPS. Of these, 813 and 586 have been identified as VUSs, respectively. 

PHTS is an autosomal dominant disease caused by germline pathogenic mutations in the *PTEN* gene. Rarely, it may also be caused by mosaicism with de novo *PTEN* mutations [36,41]. This syndrome comprises a spectrum of hamartoma conditions, including Bannayan–Riley–Ruvalcaba syndrome (BRRS), a congenital disease characterized by macrocephaly, lipomas, pigmented macules, and intestinal hamartomatous polyposis [42]; Cowden syndrome (CS), which is associated with a high risk for benign and malignant tumors of the thyroid, breast, kidney, and endometrium [36,43]; Lhermitte–Duclos disease (LDD), which is characterized by abnormal cerebellum growth [44]; segmental outgrowth-lipomatosis-arteriovenous malformation-epidermal nevus (SOLAMEN) syndrome, whose main clinical features are the presence of lipomas, hamartomatous polyps, macrocephaly, and a higher susceptibility to developing several tumors [45]; the PTEN-related Proteus syndrome (PS), which causes vascular abnormalities and various tissue overgrowths [46]; and macrocephaly-autism syndrome (MCEPHAS) [47]. Interestingly, out of 1848 *PTEN* germline mutations associated so far with PHTS diseases, 730 are VUSs (Table 1).

HMPS is characterized by multiple colorectal polyps of different histotypes (hamartomas, serrated lesions, and adenomas). The most frequent germline mutations detected in this polyposis are located in the coding and non-coding regions (upstream intron duplication) of the *GREM1* gene [48]. Currently, all four germline variants detected in this gene are classified as VUSs (Table 1). 

SPS is a rare condition defined by the occurrence of at least one of the following diagnostic criteria: (i) serrated polyp(s) in the proximal colon in a person who has a first-degree family member affected by the disease; (ii) more than five serrated polyps in the proximal colon, of which two are larger than 10 mm; and (iii) more than twenty serrated polyps [49]. Due to the low frequency of SPS cases, a driver gene has not been identified yet; however, emerging evidence suggests that germline mutations in the *RNF43* gene may be associated with this polyposis [50,51,52]. Yet, out of 111 *RNF43* germline mutations identified in patients with SPS, the vast majority (104) have been classified as VUSs (Table 1). 

### 2.2. Hereditary Nonpolyposis CRC 

Hereditary nonpolyposis colorectal cancer (HNPCC) syndromes are classified as DNA mismatch repair-deficient (MMR-d) or -proficient (MMR-p) based on the presence or absence of germline mutations in DNA MMR genes [50]. 

Lynch syndrome (LS) is an MMR-d HNPCC characterized by mutations in one or more DNA MMR genes (*MLH1*, *MSH2*, *MSH6*, and *PMS2*) [53]. These mutations have a high degree of penetrance and are thus linked to increased susceptibility to certain types of cancer [4]. An individual who has inherited a DNA MMR gene mutation faces a 70–80% chance of developing CRC during their lifetime, with this risk starting at a young age. Furthermore, women harboring genetic alterations in these genes have a significantly higher susceptibility to endometrial cancer, with a combined lifetime risk ranging from 40% to 60% [54]. The Amsterdam Criteria and Bethesda Guidelines, which are widely used for identifying individuals with LS, rely on the detection of particular site-specific malignancies that occur at an early age [4,55]. 

MMR-d cancers display marked instability at certain DNA microsatellites and are therefore classified as microsatellite instability-high (MSI-H). Additionally, these tumors are characterized by loss of expression of the affected DNA MMR protein, as determined by immunohistochemistry [53]. While CRC and endometrial cancer are the primary malignancies found in most LS families, individuals carrying these mutations also have a higher risk of developing ovarian, gastric, small intestinal, urinary tract, brain, pancreatic, and prostate cancer, as well as sebaceous neoplasms of the skin. MSI detection is based on PCR assays to amplify microsatellite sections of the DNA, followed by a comparison between normal and tumoral samples. This analysis can be used as a preliminary assessment to identify candidates for LS multigene panel testing [56]. Among the germline variants associated with LS, 828 have been identified in the *MLH1* gene (86 of which are classified as VUSs), 1732 in the *MSH2* gene (470 VUSs), 518 in the *MSH6* gene (156 VUSs), and 225 in the *PMS2* gene (61 VUSs) (Table 1). 

Approximately 50% of the patients that fulfill the Amsterdam criteria for the diagnosis of HNPCC have MMR-p disease, with no detectable germline variants in MMR genes. These individuals have a lower CRC lifetime risk compared with LS patients and are not at higher risk for malignancies other than colon cancer [57]. Currently, the only gene that could be associated with MMR-p HNPCC is *RPS20*, which encodes for a ribosomal protein. To date, five VUSs potentially associated with HNPCC have been identified in this gene (Table 1) [4].

The diagnosis of hereditary CRC syndromes is based on the classification of the identified variants in databases such as ClinVar and SIFT; thus, in silico approaches are already integrated, at least in part, into current clinical practice. However, as reported in Table 1, there is a high number of variants whose functional impact and clinical significance have not been defined yet.

## 3. In Silico Prediction of VUS Impact on Protein Function in Hereditary CRC Syndromes

In recent years, the growing repository of genetic data has led to the identification of numerous VUSs, adding further complexity to clinical decision-making. On the other hand, the identification of a myriad of genetic variants resulting from NGS studies has accelerated the development of bioinformatics tools, allowing researchers to computationally predict the functional implications of sequence variations and identify pathogenic variants [58]. Several classes of sequence variations at the nucleotide level are involved in human diseases, including substitutions, insertions, deletions, frameshifts, and nonsense mutations. Frameshift mutations and nonsense mutations are highly likely to have a detrimental impact on protein function. Therefore, the efforts of bioinformaticians have mainly focused on the development of algorithms that predict the effects of missense variants based on different approaches, such as the conservation level of amino acids at a specific position across comparable sequences or the structural impact of the amino acid change in protein stability or function [59].

In silico tools leverage computational algorithms to predict the consequences of VUSs at the molecular level. The first tools were created about twenty years ago, such as SIFT (Sorting Intolerant From Tolerant, https://sift.bii.a-star.edu.sg/index.html, latest version updated on 25 April 2024, accessed on 14 April 2024 [60,61]) and PolyPhen (Polymorphism Phenotyping, later upgraded to PolyPhen2, http://genetics.bwh.harvard.edu/pph2 version polyphen-2.2.3-databases-2021_05.tar.bz2, accessed on 14 April 2024 [62,63]). SIFT uses sequence homology and the physical characteristics of amino acids to predict whether an amino acid substitution impacts the function of the affected protein. In particular, it calculates the probability that a given amino acid substitution at a particular position will be tolerated. If the normalized value is below a specific threshold, the amino acid substitution is predicted to have a deleterious effect on protein function [62]. PolyPhen2 is more focused on predicting the potential effect of coding nonsynonymous single nucleotide polymorphisms (SNPs) based on a Bayesian probabilistic classifier with machine learning techniques and has an excellent pipeline for multiple sequence alignment [63].

SIFT and PolyPhen were used by Chao and colleagues to develop a bioinformatic algorithm named multivariate analysis of protein polymorphisms-mismatch repair (MAPP-MMR) to specifically classify pathogenic and benign *MLH1* and *MSH2* missense variants associated with LS [64].

Similarly to SIFT and PolyPhen, PROVEAN (Protein Variation Effect Analyzer; http://provean.jcvi.org/, PROVEAN v1.1, accessed on 14 April 2024 [65]) is a software that predicts whether amino acid substitutions or indels affect the biological activity of a protein. It filters sequence variants to find critical nonsynonymous or indel variants that may have deleterious effects on protein function [65].

SIFT, PROVEAN, and PolyPhen-2, together with two other tools (PhD-SNP (version PhD-SNP2.0.7, accessed on 14 April 2024) and SNPs&GO last version 8.0, accessed on 14 April 2024), were used in a comparative in silico prediction analysis to identify three *MSH6* missense mutations (G932Q, F1104Q, and E1234Q) that may contribute to protein dysfunction and CRC development [66]. In another study, Jansen and colleagues identified in silico nine predicted damaging missense variants in the *POLD1* gene by performing an integrated prediction analysis with SIFT and PROVEAN [67].

In 2011, a novel in silico prediction tool named Mutation Assessor (http://mutationassessor.org/r3/, Release 3, accessed on 14 April 2024 [68]) was created to predict the functional consequences of amino acid substitutions by considering the evolutionary conservation level of the mutated amino acid in protein homologs. This algorithm has been validated on 60,000 germline and somatic variants of diseases recorded in the OMIM database (https://www.omim.org/, version 2024, accessed on 14 April 2024), including those identified in the Cancer Genome Atlas project (https://www.cbioportal.org/, version v6.0.14, accessed on 14 April 2024). Of note, this tool was used to filter the potential pathogenetic variants in a subset of CRC patients carrying germline and somatic mutations in *APC* and *TP53* but not in other *WNT* genes (*TCF7L2*, *AMER1*, *FBXW7*, *SOX9*, *CTNNB1*). The final result of this multiple correspondence analysis was the identification of two CRC oncodriver signatures [69].

The Panther (Protein Analysis Through Evolutionary Relationships, https://www.pantherdb.org/tools, release 19.0, accessed on 14 April 2024 [70]) server is a classification system developed to provide details on the phylogeny, function, and functional impact of genetic variants that influence the evolution of protein-coding gene families. In an interesting work, Panther and other in silico tools were used to find novel pathogenetic missense variants (R358W, K306S, R310G, S433R, and R361C) in SMAD proteins, which are driver effectors of juvenile polyposis. In particular, the authors performed a comparative in silico analysis with different tools, including PANTHER, SIFT, PolyPhen, SNPs&GO, I-Mutant 3.0, and MUpro, to evaluate damaging missense variants in *SMAD* genes at both the structural and functional levels [71].

MutationTaster2 (https://www.mutationtaster.org/, version2021, accessed on 14 April 2024 [72]) is a web-based software designed to predict the potential impact of different types of genetic variants, with a particular focus on missense, intronic and synonymous variants, indel mutations, and variants in intron-exon junction regions. The MutationTaster2 predictor employs a Bayes classifier and interprets the clinical significance of the analyzed VUSs by using a comprehensive collection of SNPs from the ClinVar [73] and HGMD [74] public databases, which contain established disease variants. In a recent case report, the MutationTaster software was used to predict the functional impact of the *MLH1* frameshift mutation p.(Glu34ArgfsTer4) identified in a patient with LS. The variant was predicted to result in a non-functional protein and have a disease-causing effect [75].

Unlike other tools mentioned above, SNAP2 (Screening for Non-Acceptable Polymorphisms, http://www.ngrl.org.uk/Manchester/page/snap-screening-nonacceptable-polymorphisms.html, version 2024, accessed on 14 April 2024) [76]) does not provide predictions on the likelihood of a variant to cause a disease. Instead, it is designed to specifically assess whether the variant affects the molecular function of the protein and can thus be very helpful when combined with other prediction methods in a comprehensive computational analysis. For instance, in a recent study, SNAP2 was used together with other tools to classify as deleterious seven nonsynonymous SNPs (C76Y, C124R, C124Y, C376Y, R443C, R480W, and W487R) found in the highly conserved regions of *BMPR1A*, a gene associated with JPS [77]. 

Align-GVGD (http://agvgd.hci.utah.edu/agvgd_input.php, accessed on 23 July 2024 [78]) is one of the first free software for multiple sequence alignments. Based on the physical and chemical characteristics of amino acids, it predicts the regions that are most likely to encompass missense substitutions with deleterious or neutral effects [78]. This in silico software was used to reclassify a VUS identified in a patient with multiple colonic adenomatous polyps. The patient had the heterozygous pathogenic variant c.1187G>A (p.Gly396Asp) in exon 13 and the VUS c.1379T>C (p.Leu460Ser) in exon 14 of the *MUTYH* gene [79]. The authors reclassified the VUS as pathogenic based on the genetic evidence that it was in *trans* with the pathogenic mutation, on the clinical phenotype, and on in silico prediction findings suggesting a deleterious effect [79].

Of note, a recent in silico phylogenetic study of pathogenic variants involved in DNA repair, and therefore in CRC tumorigenesis, identified a high degree of conservation of these variants only between modern and ancient humans and not between homologous proteins of different species [80]. This evidence seems to question the validity of in silico software (e.g., SIFT, Mutation Assessor) designed for the prediction of deleterious variants based on evolutionarily conserved amino acid positions in homologous proteins. On the other hand, another recent study showed that the outcomes of functional analyses of VUSs identified in MMR genes of potential LS patients agree with the findings of in silico prediction analyses based on the conservation of residue variations in the affected DNA repair proteins [81]. Interestingly, a computational study assessed the usefulness of in silico tools to topologically map variants to surface or buried regions of highly conserved protein structures. This study confirmed that benign variants were predominantly buried inside the proteins, while pathogenic variants were mainly located on their surface [82]. Overall, this evidence suggests that in silico methods designed for identifying deleterious variants in human cancer genes based on the evolutionary conservation of variant residues may be less informative about the clinical significance of a VUS than previously thought. Nonetheless, in silico analysis of the conserved regions between homologous proteins is very useful to establish whether a given VUS maps to a domain that is conserved in different species and, therefore, is likely critical for the biological function of the affected protein.

Despite their limitations, in silico predictions offer a valuable initial screening step in VUS interpretation. Discrepancies among prediction tools emphasize the need for complementary approaches to assess VUS significance. In this regard, the accuracy of variant classification can be enhanced by integrating multiple prediction algorithms and experimental data. Various experimental methodologies can be used to ascertain whether a VUS will impact mRNA and protein stability and/or biological functions. The effects on mRNA stability and function can be investigated by low-throughput techniques such as RT-PCR, Sanger sequencing, digital droplet PCR (ddPCR), and in vitro minigene and mutagenesis assays. The effects on protein structure and stability can be assessed by different approaches, including immunohistochemistry analysis to evaluate the presence/absence of the protein in patient-derived tissues and immunoblotting analysis, which is a semiquantitative technique allowing the identification of potentially truncated proteins. On the other hand, high-throughput methodologies such as nuclear magnetic resonance (NMR) spectroscopy, X-ray crystallography, and cryo-electron microscopy (cryoEM) are essential for analyzing structural changes in the tridimensional conformation of mutated proteins [83]. The impact of a VUS on protein function can be evaluated by different in vitro methodologies, such as pull-down and enzymatic assays (if the protein is an enzyme), and by high-throughput approaches, such as mass spectrometry analysis (to assess the loss of post-translational modifications site in mutated protein) or the recently developed multiplexed (functional) assays for variant effects (MAVEs). MAVEs allow the stratification of variants by their impact and are based on a one-by-one, post hoc approach that offers an in-depth understanding of sequence-function correlations based on a versatile methodology. Indeed, MAVE experiments enable the analysis of variants in several classes of sequence, including enhancers, promoters, mRNA untranslated regions, splice sites, and in parallel in different types of proteins [84]. Overall, each of these experimental methodologies alone may be poorly informative; therefore, it is often necessary to integrate various approaches according to the VUS type, the availability of resources, equipment, and skills, and a cost-benefit assessment. Generally speaking, the main advantage of low-throughput techniques is that they are less expensive, fast, and do not require high skills; however, they sometimes do not provide sufficient insight to answer the experimental question. Conversely, high-throughput techniques are more informative but also more expensive and time-consuming. Although necessary to validate the clinical significance of a VUS, experimental approaches have limitations in terms of time and costs, thus the availability of state-of-the-art in silico functional predictors for early VUS analysis remains crucial.

Future advancements in machine learning algorithms and the incorporation of multi-omics data are anticipated to improve the reliability of in silico predictions. Currently, clinical and experimental databases have proven very useful in improving the interpretation of VUS’s impact on protein function. In Table 2, we provide a list of databases that are commonly used by clinical experts and researchers faced with the challenge of interpreting the clinical significance of a VUS.

Despite having been created several years ago, these databases are still used by the scientific community to assess the clinical and functional implications of genetic variants, especially in hereditary disorders like CRC (Table 2). Below are some significant examples of their applications in clinical and functional studies on CRC hereditary syndromes.

The authors of a recent report analyzed the occurrence of second cancers in individuals with early-onset (aged less than 50 years) LS. They provided evidence from cBioPortal annotations to show that the *FLT3* gene had the highest frequency of copy number alterations among 1438 CRC patients aged 18 to 48 years old with concomitant acute myeloid leukemia (AML). The presence of co-occurring genetic alterations in *FLT3*/*JAK2* and *JAK2*/*CTNNB1* was observed. The results provided valuable insights into the increased likelihood of AML and LS occurring together [102].

In another study, the LOVD database was employed to identify gene-phenotype associations and genotype-phenotype correlations in the *BMPR1A* gene. This information was then used to make recommendations for the clinical surveillance of JPS and modify the American College of Medical Genetics and Genomics (ACMG) classification of pathogenicity for *BMPR1A* or *SMAD4* variants associated with JPS cases [103].

Recently, a tumor mutational signature analysis conducted using the COSMIC database identified the presence of homologous recombination deficiency (HRD) in familial CRC disorders. Remarkably, this report showed that pathogenic mutations in both *BRCA1* and *RNF43* were inherited together and were associated with CRC in a family with a specific type of familial CRC known as familial colorectal cancer type X (FCCTX) [52].

Notably, the gnomAD database was recently used to assess the novel pathogenic association of a series of genes, including *NSD1*, *HDAC10*, *KRT24*, *ACACA*, and *TP63*, with CRC predisposition [104], while other databases, i.e., ClinVar, HGMD, and InSight, were previously used in a meta-analysis to identify a new pathogenic variant associated with LS in *MSH6* exon 4. In this pilot study, the authors suggested combining NGS testing and canonical MSI analysis in the diagnosis of LS in patients considered to have sporadic CRC. The inclusion criteria for NGS testing were MSI positivity, *BRAF* V600E, and *MHL1* methylation negativity [105]. 

Other computational methods developed to accurately predict the pathogenicity of a VUS, such as Multivariate Analysis of Protein Polymorphism (MAPP, http://www.ngrl.org.uk, version 3.0, [106]) and Rare Exome Variant Ensemble Learner (Revel, https://sites.google.com/site/revelgenomics, release 3 May 2021, accessed on 23 July 2024 [107]), use algorithms based on statistically multivariate analysis [106]. MAPP is a software based on the analysis of physicochemical variation in sequence alignment columns, while REVEL is an ensemble method designed to predict the pathogenicity of missense variants based on a combination of scores from 13 individual tools [107,108].

Karabachev and colleagues evaluated the accuracy of these and other computational tools (Align-GVGD, SIFT, PolyPhen2, MAPP, and REVEL) in predicting the pathogenicity of 1800 *APC* VUS reported in the NCBI ClinVar database using multiple protein sequence alignments (PMSA) of 1924 APC missense variants. When used individually, prediction accuracies for pathogenic/likely pathogenic (range 17.5–75.0%) and benign/likely benign (range 25.0–82.5%) responses differed significantly for *APC* missense variants in ClinVar. Instead, creating a curated *APC* PMSA containing >3 substitutions/site, large enough for statistically significant in silico analysis, yielded predictions of 76.2–100% accuracy with the five methods integrated into the *APC* PMSA [106]. Computational approaches based on PMSA have the potential to serve as highly effective classifiers for different variations of hereditary cancer genes. Nevertheless, several attributes of the APC gene and protein might complicate the outcomes of in silico techniques. An organized examination of these characteristics could significantly enhance the mechanization of alignment-based methodologies and the application of prognostic algorithms in genes related to hereditary cancer [106]. 

## 4. In Silico Prediction of VUS Impact on Protein Structure in CRC Hereditary Syndromes

In the last decade, great efforts have been made by researchers and bioinformaticians to develop algorithms and data sources that could help predict the effects of germline and somatic mutations on the structural stability of cancer-associated proteins. Current methodologies are primarily based on in silico structural modeling software allowing to statically or dynamically study the identified variants [83]. During a biological process, proteins can assume different conformations thanks to their intrinsic flexibility, which is crucial for acquiring their native structure. The conformation of a mutant protein differs from the native one in terms of structure and stability, altering the fine balance that regulates the functional activity of the protein [109]. 

Molecular dynamics simulation (MDS) is a widely used method for investigating the conformational dynamics of biomolecules, particularly proteins [110]. It was shown to be especially valuable for modeling alterations in the three-dimensional (3D) structures of proteins resulting from mutations such as amino acid substitutions, which modify the bonds and locations of the atoms in the wild-type protein [111,112]. MDS computes the potential energy related to the spatial coordinates of each atom in the system. The system’s potential energy is determined by evaluating a range of chemical and physical properties associated with the protein. This approach allows researchers to accurately assess the effects of a missense mutation by measuring changes in atomic or residue distances, alterations in secondary and tertiary protein structures, and modifications to hydrogen, disulfide, and ionic bonds [109]. The precision of MDS is heavily reliant on the 3D configurations of biomolecules. The use of MDS software and the analysis of established force fields have effectively uncovered the structural modifications caused by mutations, which can lead to changes in the stability of a protein, thereby affecting its biological function. The five software packages most commonly used in this area are NAMD [111], MSCALE [113], CHARMM [114], GROMACS [115], and Amber [116].

Recently, a computational approach combining in silico structural analysis and MDS was used to investigate the relationship between PHTS-associated cancer and autism spectrum disorder (ASD) by analyzing 17 selected *PTEN* mutations detected in a cohort of 138 PHTS patients. Six mutations (p.L23F, p.Y65C, p.Y68H, p.I101T, p.I122S, and p.L220V) were found exclusively in patients with ASD, six mutations were found exclusively in patients with PHTS-associated cancer (p.D24G, p.D92A, p.R130G, p.M134R, p.M205V, and p.L345V), four mutations (p.R130Q, p.C136R, p.Y155C, and p.R173C) were found in both phenotypes in different patients, and one mutation was detected in a patient with both ASD and cancer (p.S170I). The MDS analysis performed using GROMACS v4.6.3 showed that the six *PTEN* mutations detected in PHTS-associated cancer patients strongly reduce the structural stability of the protein and increase the dynamics across the domain interfaces, causing a marked tendency to protein unfolding and the closure of the active site pocket. This ultimately results in the inactivation of the enzyme [117]. 

Another important example of the application of MDS in the analysis of the structural impact of VUSs is a novel protein structure-based algorithm called deep learning-Ramachandran plot-molecular dynamics simulation (DL-RP-MDS), which was recently used to assess the structural impact of *MLH1* missense VUSs [118]. In this study, Tam and colleagues combined DL techniques with the RP-MDS method to analyze 447 *MLH1* missense VUSs. Of these, 126 were predicted to have a deleterious effect on MLH1 structure and stability [118]. The RP-MDS method combines two in silico approaches to investigate the structural changes caused by a VUS [119]. RP captures the atomic angle distortion caused by amino acid substitution, while MDS simulates the physical movement of atoms and molecules after interacting for a fixed period, and the resulting trajectories are used to determine the macroscopic thermodynamic properties of the mutated protein [119]. In addition, these data were analyzed with an unsupervised learning model consisting of an auto-encoder and neural network classifier to identify the variants resulting in significant alterations in protein structure [119]. 

Ongoing advances in the methodologies used for studying 3D protein structures, such as NMR, X-ray crystallography, and cryoEM, have significantly increased the number of known protein structures archived in the Protein Data Bank (PDB) database (https://www.rcsb.org/, latest version updated in July 2024 [120]), which currently features 218,853 recorded structures and 1,068,577 computed structure models (accessed on 7 April 2024). The consistent growth of the PDB promoted the development of various in silico prediction tools to study the structural impact of a variant based on the structure of the wild-type protein recorded in this database. Algorithms that estimate the structural impact of a single amino acid substitution can be classified into two types based on whether or not they rely on free energy calculation [83]. Energy-based methods employ experimentally determined disparities in free energy (ΔΔG) between wild-type and variant structures to develop prediction models, while non-energy-based methods directly use structural features such as variation of hydrophobicity and surface accessibility [83]. These methods can then be used to predict the resulting functional implications. In Table 3, we provided a list of in silico software commonly used to analyze the 3D structures of protein variants and their potential effects on protein stability.

Several of these software tools have been taken advantage of to improve our knowledge about the structural impact of VUSs in CRC hereditary syndromes. 

A few years ago, Doss et al. used I-Mutant 3.0, MUpro, SIFT, PolyPhen, PANTHER, and other tools to analyze the structural and functional effects of nonsynonymous SNPs in genes of the *SMAD* family. In this report, the primary mutations of SMAD native proteins, together with their amino acid locations (R358W, K306S, R310G, S433R, and R361C), were considered for structure analysis. To analyze the stability of the natural and mutant-modeled proteins, the authors used the SRide server [71]. SRide identified the stabilizing residues by calculating parameters like conservation score, stabilization center, long-range order, and surrounding hydrophobicity. The variation of potential energy and root mean square deviation values were calculated to compare the resulting native and modeled structures.

In 2022, DynaMut, DUET, and mCSM were used to predict the structural effect and the impact on gastric cancer hereditary susceptibility of a VUS (c.728G>A p.R243Q) identified in the *MSH2* gene in a Tunisian family suspected of having both hereditary diffuse gastric cancer (HDGC) and LS. Structural prediction analysis of the variant revealed that it seems to disrupt the stability of the MSH2-MLH1 complex and its binding to the DNA [142]. Further molecular modeling investigation indicated that these effects may be due to changes in the electrostatic potential of the MSH2 interaction surface. Overall, this evidence suggested that the status of the variant should be revised from VUS to likely pathogenic [142].

In another study, I-Mutant3 and MUpro were used to identify *MSH2* SNPs that could lead to structural and functional alterations resulting in CRC carcinogenesis. In particular, the authors performed a computational analysis of protein stability by integrating I-Mutant3 and MUpro support vector machine (SVM)-based algorithms. I-Mutant predicts alterations in protein stability caused by single amino acid substitutions based on the protein structure or sequence recorded in the ProTherm database. The ProTherm database comprises the most extensive and complete collection of experimental thermodynamic data. It specifically focuses on the changes in free energy resulting from mutations under various conditions and their effect on protein stability. MUpro is a machine learning-based tool that uses SVM and neural network algorithms to predict alterations in protein stability caused by individual amino acid substitutions [143]. In addition, four distinct computational tools (SIFT, PROVEAN, PANTHER, and PolyPhen) were used to predict the functional deleterious effects of *MSH2* SNPs [143]. MDS techniques revealed that six SNPs located in the MSH2/MSH6 interaction domain have a significant impact on MSH2 stability and interactions [143].

In a more recent report, a comprehensive meta-analysis based on the use of various computational software tools allowed the authors to identify pathogenic missense variants in 26 genes (*ABRAXAS1*, *ATM*, *BARD1*, *BLM*, *BRCA1*, *BRCA2*, *BRIP1*, *CDH1*, *CHEK2*, *EPCAM*, *MEN1*, *MLH1*, *MRE11*, *MSH2*, *MSH6*, *MUTYH*, *NBN*, *PALB2*, *PMS2*, *PTEN*, *RAD50*, *RAD51C*, *RAD51D*, *STK11*, *TP53*, and *XRCC2*) examined in numerous NGS panels to assess the level of hereditary risk in various cancer types, including CRC. First, the authors collected over a thousand missense variations in these genes from ClinVar and a cohort of 355 breast cancer patients. The potential effects of missense variations on protein stability were evaluated with five distinct predictor programs (SAAF2EC, MUpro, MAESTRO, mCSM, and CUPSAT). Next, the authors used the protein structures predicted by AlphaFold (AF2), an artificial intelligence (AI) system, to perform a structure-based analysis of these hereditary cancer proteins. According to previous AF2-derived findings, the confidence score for a particular variant in the AF2 structure may predict pathogenicity more reliably than any stability predictor. This study confirmed that the AF2 confidence score can be used as a valid indicator of variant pathogenicity [144]. These studies are good examples of how in silico methods can be effectively used to locate putative pathogenic variants eligible for large-scale investigations. 

In recent years, AI has proven to be a valuable tool for integrating the different in silico methods available for VUS analysis in order to expand the knowledge of VUS structure-function relationships and improve their clinical interpretation [145]. The latest advancements in AI prediction for missense variants, specifically focusing on protein structure-based approaches, highlight the complexity and the potential of this intriguing approach. Significantly, progress in protein structure prediction using deep learning, as is the case with AlphaFold2 [146] and RoseTTAFold [147], has enhanced AI models for estimating the effects of protein variants by including data on tertiary structures [83]. AlphaFold is a pioneering computational approach able to accurately predict protein structures at the atomic level, even in cases where a comparable structure is not available. RoseTTAFold (version 2.0, 2021) is an advanced software that employs deep learning techniques to rapidly and precisely predict protein structures with only a small amount of data. While ascertaining the configuration of a single protein can take several years of laboratory experimentation without the assistance of computational approaches, it can be estimated in just a few minutes using such dedicated software [148]. Importantly, these AI-based sequence and structural prediction algorithms are constantly being updated. For instance, the most recent version of Rosetta, RoseTTAFold All-Atom (RFAA), models complexes that contain proteins, nucleic acids, small molecules, metals, and covalent modifications based on their sequences and chemical structures [148]. Hopefully, in the near future, this tool will thus be integrated with an algorithm for determining the impact of genetic alterations on protein structure and function.

## 5. Conclusions

Recognizing whether a VUS is pathogenic or benign can help clinicians interpret the findings of genetic testing and provide guidance to patients and their family members who have inherited the variant. This enables a more informed clinical assessment of their “personalized” cancer risk and a better choice of follow-up options. According to recent research, cancer patients who have not responded to previous treatments might benefit from referring to multidisciplinary molecular tumor board teams [149,150]. Based on a thorough integrated review of the results of genetic testing, in silico prediction analysis, other laboratory results (imaging, pathology, biomarkers, etc.), the patient’s clinical and family history, and possibly available clinical trials, these interdisciplinary teams can then recommend tailored therapeutic solutions.

Considering that VUSs represent a high proportion of all genetic variants identified, the development of more accurate in silico predictors of their impact to support clinical surveillance decisions remains a riveting challenge [151]. The main advantage of these tools is that they provide initial insights into the potential pathogenic effect of a variant in a fast and affordable manner. Indeed, functional studies, although necessary for the classification of VUSs, cannot be considered the first approach to evaluate VUS clinical significance because they are expensive and time-consuming, which is unsustainable when dealing with rare syndromes.

In our opinion, there exists no single ideal tool capable of definitively addressing the crucial question of the possible pathogenicity of a VUS. Although different in silico tools are designed to evaluate specific effects of a VUS, in silico meta-analyses with multivariate approaches are needed to analyze multiple aspects of the clinical significance of a variant. For example, sequence-based algorithms are limited in interpreting the potential clinical significance of a VUS because they do not consider the three-dimensional structural features that determine the protein’s function. In fact, the development of AI-based algorithms that combined the structural and sequence features has significantly improved the performance of variant prediction.

However, in silico tools have limitations that can sometimes be confusing rather than clarifying. For example, different predictors can provide conflicting responses even when analyzing the same variant. This happens not only because these tools assess a variety of structural and functional characteristics (ΔΔG, conserved positions, surface or internal mutations in the three-dimensional structure of proteins, chemical alterations in secondary and tertiary structures, etc.) but also because their algorithms are frequently designed using inaccurate benchmarks. Currently, bioinformaticians who develop a novel prediction tool assess the performance of their new software by comparing it with previously published predictors using consolidated variant databases. This frequently introduces bias because predictor performance is evaluated with the same data used to create the tool. Thus, strong and impartial benchmarking by independent groups is necessary to develop more accurate tools [145].

Massive advancements have been achieved in the computational prediction of the structural and functional impact of genetic variants in recent years. Prediction tools offer a scalable and quick way for clinical and research laboratories to assess the potential effects of novel variants. However, determining to what extent clinicians can trust the findings from in silico prediction methods is still a challenging task. According to ACMG guidelines, the specificity of most in silico tools is rather low, which affects their reliability when it comes to predicting missense changes with a milder effect and causes missense variants to be overpredicted as deleterious [152]. While computational prediction methods alone are insufficient to ascertain the pathogenicity of a variant, they are very useful in selecting the VUSs that warrant experimental characterization to validate their clinical significance, especially for VUSs detected in patients (or their relatives) with hereditary CRC syndrome phenotypes. 

**Figure 1:**
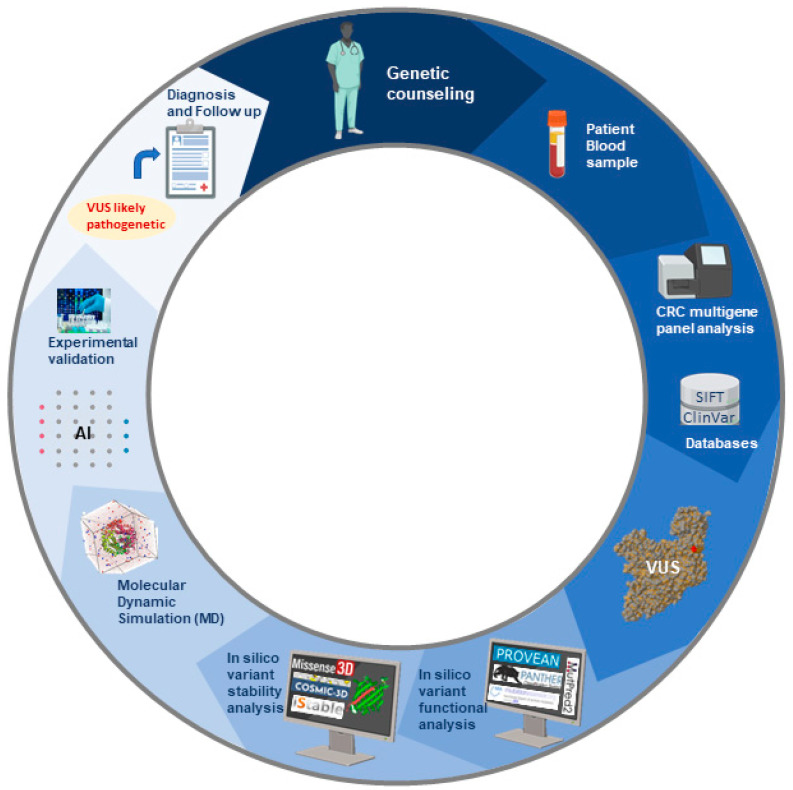
Schematic workflow to assess the clinical significance of a VUS. The process involves an initial in silico prediction analysis of the structural and functional effects of the variant and a final experimental validation to achieve a more personalized diagnosis and follow-up program.

Furthermore, these tools are usually based on complicated algorithms that are difficult to handle for non-experts, and their use is hindered by the difficulty of correctly interpreting the results. A further limitation to their application in clinical routine is the so-called data circularity [153]. Grimm and collaborators defined two types of circularities that can distort the evaluation of predictor tools. Type 1 circularity mostly impacts techniques that are based on machine learning. A technique is vulnerable to type 1 circularity when it reuses training data for the model in the validation of its execution. Type 2 circularity arises when the same datasets of protein variants are used for the training and evaluation of the tools employed for predicting the clinical significance of a VUS. This may lead to misleading conclusions on the predictive ability of the algorithms in the study of proteins that have an equal number of pathogenic and benign variants, potentially resulting in inaccurate predictions [153]. In particular, predictors are frequently tested for effectiveness using extensive datasets containing confirmed deleterious or benign genetic variations. The benchmarking data may overlap with the data used to train certain supervised predictors, resulting in data reuse or circularity. This, in turn, can lead to an overestimation of the performance and effectiveness of such predictors [145]. Large-scale functional tests known as deep mutational scans offer a possible solution to the problem of circularity by providing independent datasets of variant effect measurements. Such functional tests appear more reliable in predicting the clinical impact of mutations [145]. In addition, the remarkable developments made in protein structure prediction and MDS techniques not only demonstrate the potential benefits of AI in structural biology but also open new promising horizons in AI-assisted structural and functional studies of genetic variants, especially VUSs. As shown by the growing number of articles published on this topic, MDS and structural-functional predictors are becoming crucial for the assessment of the functional and clinical impact of VUSs. Previous reports have demonstrated that these integrated approaches are feasible and provided hints for creating learning models with even more accurate variant effect prediction capabilities despite also highlighting a variety of issues [83]. Predictive structural AI-based methodologies also have the potential to overcome the main limitations of in silico tools in VUS evaluation, allowing the development of increasingly personalized clinical management strategies for patients (Figure 1). A broader application of AI-based structure prediction tools for protein function analysis may accelerate the assessment of the clinical impact of a variant by reducing the time and number of experiments needed to confirm it. As a result, current research is focused on creating new algorithms designed to model protein structures and predict within a single in silico pipeline the structural and functional effects of VUSs, thereby allowing a more accurate interpretation of their clinical implications.

## Figures and Tables

**Table 1:** Number of driver genetic variants identified in hereditary CRC syndromes as reported in the ClinVar Miner database (https://clinvarminer.genetics.utah.edu/, accessed on 7 April 2024 ClinVar version 2024-03-31, [28]).

Hereditary CRC Syndrome	Driver Genes	Pathogenic	Likely Pathogenic	Uncertain Significance	Likely Benign	Benign	Total
FAP and AFAP	*APC*	1796	213	6139	2249	228	10,625
MAP	*MUTYH*	9	1	14	27	0	51
PPAP	*POLD*	4	2	2378	1732	98	4214
	*POLE*	16	12	379	231	60	698
NTHL1 tumor syndrome	*NTHL1*	58	38	102	3	0	201
PJS	*STK11*	193	55	837	750	77	1912
JPS	*BMPR1A*	164	41	813	552	30	1600
	*SMAD4*	139	37	586	569	17	1348
PHTS	*PTEN*	487	122	730	456	53	1848
HMPS	*GREM1*	0	0	4	0	0	4
SPS	*RNF43*	2	2	104	2	1	111
LS	*MLH1*	527	120	86	40	55	828
	*MSH2*	756	255	470	165	86	1732
	*MSH6*	203	55	156	57	47	518
	*PMS2*	83	32	61	13	36	225
MMR-p HNPCC	*RPS20*	0	0	5	0	0	5

**Table 2:** List of major databases for analyzing the clinical significance of genetic variants. The resources are listed in alphabetical order.

Database	Description	Link	References	Number of Tool Citations *
ActiveDriverDB	Human proteo-genomics database that annotates disease mutations and population variants using post-translational modifications	https://activedriverdb.org/ (accessed on 14 April 2024)	[85]	3
cBioPortal (Cancer Genomics Portal)	Open-access resource that is useful to interactively explore multidimensional cancer genomics data sets. It presently provides access to data from about 100,000 tumor samples collected from 218 different cancer research studies	https://www.cbioportal.org/ (accessed on 14 April 2024)	[86]	2113
ClinVar (Clinical Variants)	Portal of human variations classified for diseases	https://www.ncbi.nlm.nih.gov/clinvar/ (accessed on 14 April 2024)	[73]	1055
ClinVar Miner (Clinical Variants Miner)	Portal for viewing and filtering ClinVar data	https://clinvarminer.genetics.utah.edu/ (accessed on 14 April 2024)	[28]	4
COSMIC (Catalogue Of Somatic Mutations In Cancer)	Curated database of somatic and germline mutations	https://cancer.sanger.ac.uk/cosmic (accessed on 14 April 2024)	[87]	212
dbNSFP (Database for Nonsynonymous SNPs’ Functional Predictions)	Database of functional predictions and annotations for human nonsynonymous SNPs	http://database.liulab.science/dbNSFP#database (accessed on 14 April 2024)	[88]	35
dbSNP (Single Nucleotide Polymorphism Database)	SNP catalog designed to facilitate large-scale studies and association between genetics, functional implications, population genetics, and evolutionary biology of SNPs	https://www.ncbi.nlm.nih.gov/snp/ (accessed on 14 April 2024)	[89]	1087
dbVar (Database of Genomic Variation)	Repository of structural variations in the human genome allowing to search, read, and download data from submitted studies	https://www.ncbi.nlm.nih.gov/dbvar/ (accessed on 14 April 2024)	[90]	27
DoCM (Database Of Curated Mutations)	Curated database of validated cancer driver mutations	http://www.docm.info/ (accessed on 14 April 2024)	[91]	16
GnomAD (GeNOMe Aggregation Database)	Collection of standardized exome and genome sequencing data from numerous large-scale sequencing initiatives	https://gnomad.broadinstitute.org/, accessed on 14 April 2024	[92]	866
HGMD (The Human Gene Mutation Database)	Comprehensive repository of inherited mutation data for medical research, genetic diagnosis, and NGS studies	https://www.hgmd.cf.ac.uk/ac/index.php/, accessed on 14 April 2024	[93]	225
InSiGHT (International Society for Gastrointestinal Hereditary Tumours)	Extensive database of DNA variations that have been re-sequenced in genes associated with gastrointestinal cancer	https://www.insight-group.org/variants/databases/ (accessed on 14 April 2024)	[94]	62
LoVD (Leiden Open Variation Database)	Web-based open-source database collecting DNA sequence variants associated with genetic (hereditary) diseases	https://www.lovd.nl/ (accessed on 14 April 2024)	[95]	158
OMIM (Online Mendelian Inheritance in Man)	Collection of genetic phenotypes associated with Mendelian inherited disorders	https://omim.org/ (accessed on 14 April 2024)	[96]	8182
PharmGKB (Pharmacogenomics Knowledge Base)	Comprehensive database providing researchers and clinicians with information regarding how genetic diversity affects drug response	https://www.pharmgkb.org/ (accessed on 14 April 2024)	[97]	546
SNPedia (Single Nucleotide Polymorphism encyclopedia)	Database referencing peer-reviewed scientific literature that gathers data on the impact of DNA polymorphisms with an emphasis on medical, phenotypic, and genealogical correlations of SNPs	https://www.snpedia.com/index.php/SNPedia (accessed on 14 April 2024)	[98]	19
UMD (Universal Mutation Database)	Database of driver mutations, focusing on their importance for the twelve main types of cancer	https://bio.tools/umd (accessed on 14 April 2024)	[99]	15
VarSite (Variant Site database)	Web service that maps natural variations from gnomAD and known disease-associated variants from UniProt and ClinVar onto 3D protein structures stored in the Protein Data Bank	https://www.ebi.ac.uk/thornton-srv/databases/VarSite (accessed on 14 April 2024)	[100]	4
VIPdb (Variant Impact Predictor Database)	Comprehensive resource that facilitates the exploration of suitable tools and aids in the creation of enhanced methods for accurately predicting the impact of genetic variants	https://genomeinterpretation.org/vipdb (accessed on 14 April 2024)	[101]	3

* Based on a PubMed search performed using the name or URL link of the tools as keywords (accessed July 2024).

**Table 3:** List of useful in silico prediction algorithms for predicting the impact of variations on protein structure and stability. The resources are listed in alphabetical order.

Resource	Description	Link	References	Number of Tool Citations *
AUTO-MUTE version 2.0	Software using ΔΔG calculations and knowledge-based potentials	http://proteins.gmu.edu/automute (accessed on 20 April 2024)	[121]	5
Cosmic-3D Release v99	Tool that analyzes cancer mutations within the framework of three-dimensional protein structures	https://cancer.sanger.ac.uk/cosmic3d/ (accessed on 20 April 2024)	[122]	4
CUPSAT	Software using ΔΔG calculations with mean force atom pair and torsion angle potentials	https://cupsat.brenda-enzymes.org/ (accessed on 20 April 2024)	[123]	34
DynaMut	Software using ΔΔG calculations to predict the effects of variants on protein flexibility	http://biosig.unimelb.edu.au/dynamut/ (accessed on 20 April 2024)	[124]	47
DUET	Software that predicts the effects of mutations on protein stability by calculating changes in ∆∆G	https://biosig.lab.uq.edu.au/duet (accessed on 20 April 2024)	[125]	11
FOLD-X Version 3.0	Software using empirical force fields to calculate ΔΔG	https://software.embl-em.de/software/6 (accessed on 20 April 2024)	[126]	39
i-Mutant 3.0	Software using support vector machines (SVMs) to calculate ΔΔG	http://gpcr2.biocomp.unibo.it/cgi/predictors/I-Mutant3.0/I-Mutant3.0.cgi (accessed on 20 April 2024)	[127]	27
iStable Version 2.0	Software using SVMs to analyze protein stability and calculate ΔΔG	http://predictor.nchu.edu.tw/iStable (accessed on 20 April 2024)	[128]	37
MAESTRO Version 1.2.35	Software using ΔΔG calculations and multi-agent stability prediction	http://biwww.che.sbg.ac.at/MAESTRO (accessed on 20 April 2024)	[129]	62
mCSM	Software using graph-based signatures to calculate ΔΔG	https://biosig.lab.uq.edu.au/mcsm (accessed on 20 April 2024)	[130]	137
Missense3D (Release June 2019)	Tool that predicts structural alterations resulting from amino acid substitutions. Analysis of experimental coordinates and expected structures is also possible	http://missense3d.bc.ic.ac.uk/missense3d/ (accessed on 20 April 2024)	[131]	21
MUpro (Release 6.0, 2021)	Software using SVMs to predict variation in protein stability	http://mupro.proteomics.ics.uci.edu/ (accessed on 20 April 2024)	[132]	86
Mutfunc Version 2.0	Web resource reporting mutations that are either expected to cause instability in protein structure or that occur in functionally significant regions	www.mutfunc.com (accessed on 20 April 2024)	[133]	2
NeEMO	Software using amino acids involved in protein-to-protein interaction networks to calculate ΔΔG	https://biocomputingup.it/ (accessed on 20 April 2024)	[134]	18
Phyre 2 version 2.0	Tool that predicts protein sequence structure and function using automatic fold recognition	http://www.sbg.bio.ic.ac.uk/~phyre2/html/page.cgi?id=index (accessed on 20 April 2024)	[135]	191
PhyreRisk Version 1.0.1	Open-access program that maps human variations onto protein structure, integrating genomic, proteomic, and structural data	http://phyrerisk.bc.ic.ac.uk/ (accessed on 20 April 2024)	[136]	3
PMut Version 2017	Software designed to identify and predict pathological mutations. It labels mutations by processing several types of sequence information using neural networks	https://mmb.irbbarcelona.org/PMut (accessed on 20 April 2024)	[137]	183
ProMaya	Software using random forests regression for ΔΔG calculations	http://bental.tau.ac.il/ProMaya/ (accessed on 20 April 2024)	[138]	3
SAAFEC-SEQ Version 1.0	Software using multiple linear regression to calculate ΔΔG	http://compbio.clemson.edu/lab/ (accessed on 20 April 2024)	[139]	7
SRide	Server allowing for detection of stabilizing residues within proteins	http://sride.enzim.hu (accessed on 20 April 2024)	[140]	7
STRUM Version STRUM.tar.bz2	Software that predicts alterations caused by single-point nonsynonymous SNPs in protein folding stability by calculating changes in ∆∆G	https://zhanggroup.org/STRUM/ (accessed on 20 April 2024)	[141]	3

* Based on a PubMed search performed using the name or URL link of the tools as keywords (accessed July 2024).

## Data Availability

The authors reported the link to access to each dataset, software, and server mentioned in this work.
